# Functionalizable Antifouling Coatings as Tunable Platforms for the Stress-Driven Manipulation of Living Cell Machinery

**DOI:** 10.3390/biom10081146

**Published:** 2020-08-05

**Authors:** Ivana Víšová, Barbora Smolková, Mariia Uzhytchak, Markéta Vrabcová, Djamel Eddine Chafai, Milan Houska, Matěj Pastucha, Petr Skládal, Zdeněk Farka, Alexandr Dejneka, Hana Vaisocherová-Lísalová

**Affiliations:** 1 Institute of Physics CAS, Na Slovance 1999/2, 182 21 Prague, Czech Republic; visova@fzu.cz (I.V.); smolkova@fzu.cz (B.S.); uzhytchak@fzu.cz (M.U.); vrabcova@fzu.cz (M.V.); chafai@fzu.cz (D.E.C.); houska@fzu.cz (M.H.); dejneka@fzu.cz (A.D.); 2 Department of Biochemistry, Faculty of Science, Masaryk University, Kamenice 5, 625 00 Brno, Czech Republic; mpastucha@gmail.com (M.P.); skladal@chemi.muni.cz (P.S.)

**Keywords:** zwitterionic material, cell mechanotransduction, cell signaling, functional biointerfaces, antifouling polymer brushes, surface modification

## Abstract

Cells are continuously sensing their microenvironment and subsequently respond to different physicochemical cues by the activation or inhibition of different signaling pathways. To study a very complex cellular response, it is necessary to diminish background environmental influences and highlight the particular event. However, surface-driven nonspecific interactions of the abundant biomolecules from the environment influence the targeted cell response significantly. Yes-associated protein (YAP) translocation may serve as a marker of human hepatocellular carcinoma (Huh7) cell responses to the extracellular matrix and surface-mediated stresses. Here, we propose a platform of tunable functionable antifouling poly(carboxybetain) (pCB)-based brushes to achieve a molecularly clean background for studying arginine, glycine, and aspartic acid (RGD)-induced YAP-connected mechanotransduction. Using two different sets of RGD-functionalized zwitterionic antifouling coatings with varying compositions of the antifouling layer, a clear correlation of YAP distribution with RGD functionalization concentrations was observed. On the other hand, commonly used surface passivation by the oligo(ethylene glycol)-based self-assembled monolayer (SAM) shows no potential to induce dependency of the YAP distribution on RGD concentrations. The results indicate that the antifouling background is a crucial component of surface-based cellular response studies, and pCB-based zwitterionic antifouling brush architectures may serve as a potential next-generation easily functionable surface platform for the monitoring and quantification of cellular processes.

## 1. Introduction

Living cells are continuously sensing their microenvironment and adapt to different physicochemical events during their lifetime [1,2]. Besides soluble chemical signals, mechanical cues influence cells persistently over time, involving the activation or inhibition of different signaling pathways [3]. Mechanotransduction, defined as the conversion of mechanical cues into biochemical signals, leads to cellular responses important for cell function in health (i.e., growth and differentiation) and diseases (i.e., cancer malignant progression) [4]. Among these mechanosensing processes, integrin mediates cell adhesion, leads to the cell response by recruiting intracellular multiprotein assemblies such as F-actin [5], or influences the Yes-associated protein (YAP) location [6].

YAP is a transcriptional regulator and oncoprotein, tightly controlled by different regulatory systems sensitive for the extracellular matrix and microenvironment [7,8]. In an inactive form, YAP is found mostly in the cytoplasm; when activated, it concentrates in the nucleus to operate the transcription of genes involved in cell division or apoptosis [7]. The regulation of YAP is a complex process, which encompasses several different factors, such as cell density and polarity, mechanical stress, cellular energy status, etc. [9,10]. In detail, the subcellular location of YAP is influenced by the substrate rigidity and topography of the surface [11,12,13], cell geometry and morphology driven by the surface characteristics and surrounding conditions [14,15,16], or the density of the population and contact among the cells [17,18].

Surface-based methods are widely used to study cell adhesion and migration. However, surfaces engineered to accurately tailor cell-surface interactions are increasingly needed [19,20]. Recent works focused on the prevention of cell adhesion noticed antifouling coatings as useful platforms to diminish adhesion [21,22,23] and/or to pattern cell growth [24,25]. Well-defined antifouling coatings with minimum backgrounds of nonspecific bindings, even from complex biological media, represent an attractive approach to elucidate the effects of surface physicochemical properties on cellular behaviors. Surface-based platforms for specific targeting should be rationally designed to (i) integrate functional groups for specific cell receptors targeting, (ii) suppress nonspecific interactions, and (iii) use integrative markers for the assessment of the cell behavior in response to the surface binding. In some portion of the works, the antifouling background was decorated with an arginine, glycine, and aspartic acid (RGD) moiety containing peptides (R: arginine, G: glycine, and D: aspartic acid and further RGD peptides). The RGD peptides are well-known to promote cell adhesion through specific integrin interactions [26]. For example, the study on the effect of the osteogenic differentiation of mesenchymal stem cells proved the possibility to modulate differentiation by using antifouling carboxybetaine-based hydrogel coating functionalized with RGD peptides (RGD-functionalized coatings) [27]. Using a functionalized 3D fiber scaffold covered with antifouling brushes, the authors in [28] showed that cell attachments and spreading are dependent on the RGD moiety surface concentration.

In this work, we demonstrate a smooth approach to control the level of extracellular mechanical stress using the cell adhesion-promoting RGD-functionalized antifouling polymer brushes. To define the level of mechanical stress, we monitored the colocalization of YAP in the cell nucleus. Two distinct types of state-of-art functionable antifouling coatings based on pCB zwitterionic brushes [29,30,31] were employed and compared. First, a set of RGD-functionalized homopolymers poly(carboxybetaine acrylamide) (pCBAA) was prepared by employing different concentrations of the RGD peptide during the immobilization. Second, a set of RGD-functionalized random copolymers of carboxybetaine methacrylamide (CBMAA) and nonionic poly(*N*-(2-hydroxypropyl) methacrylamide) HPMAA (p(CBMAA-*ran*-HPMAA)) with different molar ratios of functionable CBMAA was prepared. Standard carboxy- or a mix of carboxy-/hydroxy-functional oligo(ethylene glycol) (OEG)-based self-assembled monolayer (SAM) [32,33] commonly used to enhance the resistance of surfaces to fouling were functionalized with the RGD peptide to provide reference systems for comparison of the effects of antifouling properties on mechanotransduction studies.

## 2. Materials and Methods

### 2.1. Reagents

The buffer solutions were prepared using ultrapure water (18.0 MΩ·cm, Milli-Q^®^ system, Merck, Darmstadt, Germany). Phosphate-buffered saline (PBS, 0.01-M sodium phosphate, 0.138-M sodium chloride, 0.0027-M potassium chloride, pH 7.4) and borate buffer (10-mM sodium borate, pH 8.5) were prepared from a stock solution from Sigma-Aldrich, St. Luis, MO, USA. The PBS-NaCl buffer (0.01-M phosphate, 0.75-M sodium chloride, 0.0027-M potassium chloride, pH 7.4) was prepared from PBS stock solution. The initiator ω-mercaptoundecylbromoisobutyrate was from Prochimia, Gdansk, Poland. The carboxybetaine acrylamide, carboxybetaine methacrylamide, and *N*-(2-hydroxypropyl) methacrylamide monomers were from Specific Polymers, Castries, France. 1,4,8,11-tetramethyl-1,4,8,11-tetraazacyclotetradecane (Me_4_Cyclam, 98%), CuCl (≥99.995%), CuCl_2_ (99.999%), methanol (≥99.9%), and ethanol (99,9%) were from Sigma-Aldrich. Tetrahydrofuran (THF, ≥99.9%) was from Penta, Prague, Czech Republic. The cell adhesion-promoting peptide (RGD peptide) with a sequence of H-RRRGGGGRGDSP-OH (12 residues), purity >98%, was synthesized by Pepscan, Lelystad, The Netherlands. *N*-hydroxysuccinimide (NHS) and *N*-ethyl-*N*’-(3-diethylaminopropyl)carbodiimide (EDC) were purchased from AP Czech, Prague, Czech Republic. 2-(2-aminoethoxy)acetic acid (AEAA) was purchased from VWR International, Radnor, PA, USA. The following fluorescent probes and antibodies were purchased from Thermo Fisher Scientific (Waltham, MA, USA): CellMask™ Green for the cell membrane, propidium iodide for the cell death assessment, Hoechst 33,342 for nuclear staining, Alexa Fluor 568 secondary anti-rabbit antibody, and Alexa Fluor™ 488 Phalloidin (1:500) (A12379)). Anti-YAP rabbit antibody (14074S) was purchased from Cell Signaling Technology, Ledien, Netherlands.

### 2.2. Preparation of RGD-Functionalized Coatings

For all experiments, we used rationally designed the RGD moiety with a sequence of H-RRRGGGGRGDSP-OH (12 residues), containing a primary amine-rich sequence (RRR) at its N-terminus and a cell adhesion-promoting sequence (RGD) near its C-terminus. Thus, the peptide can be easily immobilized on the surface without substantially limiting its cell adhesion-promoting activity. The immobilization of the RGD peptide to the surfaces was performed using amine coupling chemistry via the covalent binding of primary amines with activated carboxy groups of the surface coatings.

#### 2.2.1. Preparation of pCB-Based Polymer Brushes

pCB brushes were prepared by surface-initiated atom transfer radical polymerization (SI-ATRP), as described elsewhere [31], and their structures were checked by infrared spectroscopy (data not shown). After polymerization, all coatings were stored in PBS. Before functionalization, coatings were rinsed with ultrapure water and sterilized in 70% ethanol for 30 min. All further steps were carried out under strictly sterile conditions using sterile solutions. After sterilization, coatings were immersed in ultrapure water for 5 min and subsequently activated with a mixture of 0.1-M NHS and 0.5-M EDC for 25 min. Afterwards, the coatings were shortly washed with ultrapure water, and the solution of the RGD peptide in borate buffer (10 mM, pH 8) was added and reacted for 20 min. For functionalization of the pCBAA coating, different concentrations of the RGD peptide were used (0, 1, 10, 50, 100, and 500 μg/mL). For immobilization of the RGD peptide on both copolymers, the concentration of 850 μg/mL was applied. After immobilization, all coatings were rinsed with water and immersed in the deactivation solution of 1-M aminoethoxy acetic acid (AEAA) for 30 min. Finally, all the coatings were rinsed with PBS-NaCl and stored in sterile PBS until use.

#### 2.2.2. Preparation of the Carboxy-Functional OEG-Based Self-Assembled Monolayer (OEG-SAM)

Gold-coated glass substrates were rinsed with ultrapure water and isopropyl alcohol and sonicated in isopropyl alcohol for 30 min. Afterwards, the chips were immersed in an ethanol solution of HS-(CH_2_)_11_-(EG)_6_-OCH_2_-COOH and HS-(CH_2_)_11_-(EG)_4_-OH in different molar ratios (0:100, 35:65, 65:35, and 100:0, respectively), with the total concentration of 1 mM of thiol groups, and left for 3 days at room temperature in the dark to form a SAM with different contents of carboxy and hydroxy groups. All further steps were held under strictly sterile conditions using sterile solutions. The coatings were rinsed with ethanol, left in ultrapure water for 10 min, and, subsequently, activated with a mixture of 0.025-M NHS and 0.125-M EDC for 30 min. Afterwards, the coatings were shortly washed with ultrapure water, and a solution of the RGD peptide in 10-mM sodium acetate buffer (pH 5.0) was added and left to react for 12 min. For pure HS-(CH_2_)_11_-(EG)_6_-OCH_2_-COOH coatings, solutions of different concentrations of RGD peptides were used (0, 0.1, 1, 50, and 100 μg/mL) for functionalization. For the immobilization of the RGD peptide on the mixed SAM coatings, a concentration of 50 μg/mL was applied. After immobilization, all coatings were subsequently immersed in sodium acetate buffer, PBS-NaCl, sodium acetate, 1-M ethanolamine, and sodium acetate, respectively, each for 5 min. Then, all the coatings were stored in sterile PBS until use.

### 2.3. Surface Characterization by SPR

For the characterization of the functionalization (more details in the Supplementary Materials), a six-channel spectroscopic SPR sensor developed at the Institute of Photonics and Electronics (Prague, Czech Republic) combined with a dispersionless microfluidic system equipped with temperature controller was used. For this sensor at a resonant wavelength around 750 nm, a 1-nm SPR wavelength shift represents a change in the surface protein concentration of 17 ng/cm^2^ [2]. Antifouling properties were confirmed using an angular MP-SPR with a 4-channel microfluidic system, equipped with LED sources of 670 nm and 785 nm wavelengths (BioNavis 400 KONTIO, BioNavis, Tempere, Finland). To transform the shift in the SPR resonance angle to mass units of unspecifically adsorbed proteins, a value of 0.001~0.85 ng/cm^2^ at 785 nm was used. More details on the SPR experiments can be found in the Supplementary Materials, including the representative SPR sensorgrams for surface functionalation with the RGD-\ peptide (Figure S1), as well as the obtained immobilization values (Figure S2).

### 2.4. Cell Culture

Human hepatocellular carcinoma cell lines Huh7 obtained from the Japanese Collection of Research Bioresources (JCRB) were cultured in EMEM medium (American-Type Culture Collection, ATCC, Manassas, VA, USA) supplemented with 10% fetal bovine serum (FBS, Thermo Fisher Scientific) as recommended by the supplier. Cells were cultivated at 37 °C in a humidified atmosphere containing 5% CO_2_, and the medium was changed once a week. The Huh7 cell line was selected as a model system due to its high susceptibility to external physicochemical stresses.

### 2.5. Assessment of Cell Death

Propidium iodide (PI) was used for the cell death assessment. Huh7 cells were seeded on the sterilized substrates at an initial density of 25,000 cells/cm^2^ and were grown under standard conditions for 72 h. Cells were afterwards labeled with 1-mL PI (50 μg/mL) and Hoechst 33,342 as nucleus staining for 5 min in the dark at room temperature. After staining, labeled cells were imaged using spinning disk confocal microscopy (IXplore SpinSR Olympus, Tokyo, Japan)). ImageJ software (National Institutes of Health, NIH, Bethesda, MD, USA) was used for image processing and quantification. PI-stained cells were considered to be dead cells. Cells treated with 20% ethanol for 30 min were used as the positive control.

### 2.6. Localization and Quantification of Endogenous YAP

Huh7 cells were seeded on the sterilized substrates at an initial density of 25,000 cells/cm^2^ and were grown under standard cell culture conditions. Control cells were seeded onto standard 35-mm Petri dishes (Cellvis, Sunnyvale, CA, USA). After 72 h, the cells were washed with PBS and fixed with 4% paraformaldehyde for 10 min at room temperature. After fixation, the samples were washed with PBS and permeabilized with 0.5% Triton-X 100 in PBS for 20 min at room temperature (RT). Post fixation and permeabilization, cells were incubated with diluted primary anti-YAP antibody (1:500) for 1 h at room temperature. After washing with PBS, the cells were incubated with goat anti-rabbit Alexa Fluor 568 secondary antibody diluted in PBS (1:1000) for 1 to 2 h at room temperature in the dark. Alexa Fluor™ 488 Phalloidin diluted in PBS (1:500) was used sequentially in order to visualize F-actin in cells. Hoechst 33,342 was used as nucleus staining. Post staining, labeled cells were imaged using spinning disk confocal microscopy (Spin SR, Olympus). ImageJ software (NIH) was used for image processing and quantification.

### 2.7. Spinning Disk Confocal Microscopy

High-resolution spinning disk confocal microscopy (Spin SR, Olympus) was used for Huh7 cell visualizations. Huh7 cells were grown on different types of substrates for 72 h and stained with Hoechst 33,342 for nuclear staining in blue, YAP antibody in red, and Alexa Fluor™ 488 Phalloidin was used in order to visualize F-actin in cells in green. Fluorescence images were taken with the acquisition software cellSens (Olympus) and processed using ImageJ.

### 2.8. Statistical Analysis

Data obtained from independent experiments are presented as the mean ± standard error of the mean (SEM). Statistical analysis was determined using a one-way analysis of variance and the Newman-Keuls test. Statistical analysis was performed using MaxStat Pro 3.6 (MaxStat Software, Jever, Germany). The differences were considered statistically significant at *p* < 0.05.

## 3. Results

### 3.1. Antifouling Properties of RGD-Functionalized Coatings

**Figure 1:**
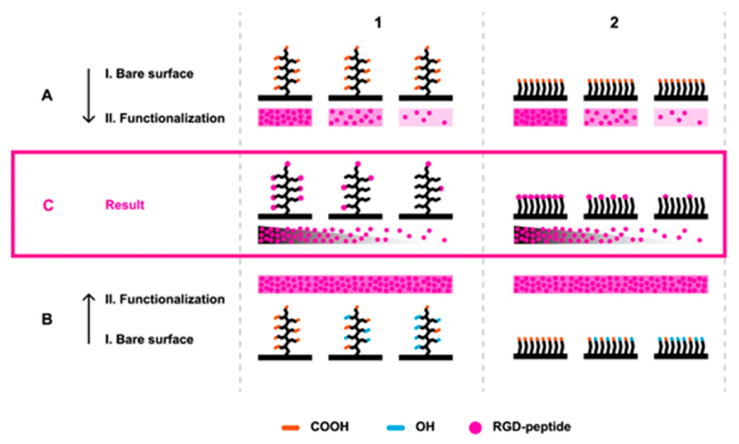
Immobilization strategies. Arginine, glycine, and aspartic acid (RGD)-functionalized sets of poly(carboxybetain) (pCB) coatings (**C1**) and oligo(ethylene glycol) self-assembled monolayer (OEG SAM) coatings (**C2**) were prepared using two different approaches. First (**A**), only carboxy-functional coatings were activated and exposed to solutions of RGD peptides with different concentrations. The second (**B**) set of coatings with different ratios of functionalizable carboxy groups in relation to nonfunctionalizable hydroxy groups was prepared, activated, and exposed to a high concentration of RGD peptides. The functionalization approaches of A and B were performed on pCB homopolymer and copolymer brushes (**1**) and OEG-based carboxy-functional homogenous and mixed SAM (**2**) coatings.

A set of pCB brush coatings and OEG-based SAM coatings functionalized with variable concentrations of RGD peptides were prepared to investigate the effects of the RGD peptide-decorated antifouling background on mechanotransduction cell signaling. Based on our previous study [31], we used optimized biocompatible and highly hydratable antifouling pCB brush coatings with wet thicknesses around ~80 nm (characterized by spectroscopic ellipsometry; data not shown). Two different approaches to the preparation of the coatings functionalized with varying RGD concentrations were employed as follows: first, only carboxy-functional coatings of pCBAA or SAM of HS-(CH_2_)_11_-(EG)_6_-OCH_2_-COOH were RGD-functionalized using immobilization solutions of RGD peptides of different concentrations (Figure 1A1,A2). Second, a gradient of functionalizable elements (Figure 1C1,C2) was prepared by the copolymerization or mixing of different ratios of carboxy-functional and hydroxy-functional components for pCB brushes or OEG SAM coatings, respectively. In the latter case, the concentration of RGD peptides was specified for each type of the coating to be high enough to saturate the activated groups (Figure 1B1,B2).

Depending on the cell line, the first cells can already sense and initiate the adhesion process after 1 h of incubation [1]. Therefore, antifouling properties of RGD-functionalized pCB or OEG SAM coatings were tested (i) after incubation in cell growth medium for 60 min and (ii) after incubation in undiluted human blood plasma for 10 min, as this is a commonly used antifouling characterization method [33]. While in the cell growth medium, the antifouling pCB coatings showed a fouling level under the limit of detection, and OEG SAM showed a fouling level up to 35 ng/cm^2^. RGD peptide concentrations used for the functionalization of both types of carboxy-functional coatings did not have any influence on the fouling level from the cell growth medium (Table 1). To confirm the antifouling properties of all pCB coatings, the coatings were exposed to undiluted human blood plasma. The level of fouling on pCB coatings before the activation of carboxy groups remained below 10 ng/cm^2^. The fouling level after the RGD functionalization was around 27 ng/cm^2^, which is consistent with the previously published data on decreased resistance following the functionalization of antifouling polymer brushes [34]. Fouling from undiluted blood plasma is neither dependent on the volume concentrations of RGD peptides nor the content of carboxy groups in functionalized copolymer coatings (Figure S3). The fouling resistance to the undiluted blood plasma of OEG-based SAM coatings was ~150 ng/cm^2^. These values were ~five times lower compared to the pCB-based coatings. 

### 3.2. Modulation of Mechanotransduction Signaling

Hepatocytes are very sensitive cells [35,36,37], which makes them a good model system for mechanotransduction-signaling studies. Recently we showed that nonfunctionalized antifouling polymer brush coatings pCBAA, pCBMAA, pHPMAA, and their copolymers are not cytotoxic for Huh7 cells [31]. Here, we confirmed no cytotoxicity, even for activated and deactivated or RGD-functionalized pCB antifouling coatings. The cell death ratios of Huh7 cells for all tested coatings remained around 3% after three days of cultivation, which is the same as the cell death ratio of the control (Figure S4). Apparently, the majority of cells cultivated with RGD-functionalized pCB brushes remained alive after three days of incubation.

**Figure 2:**
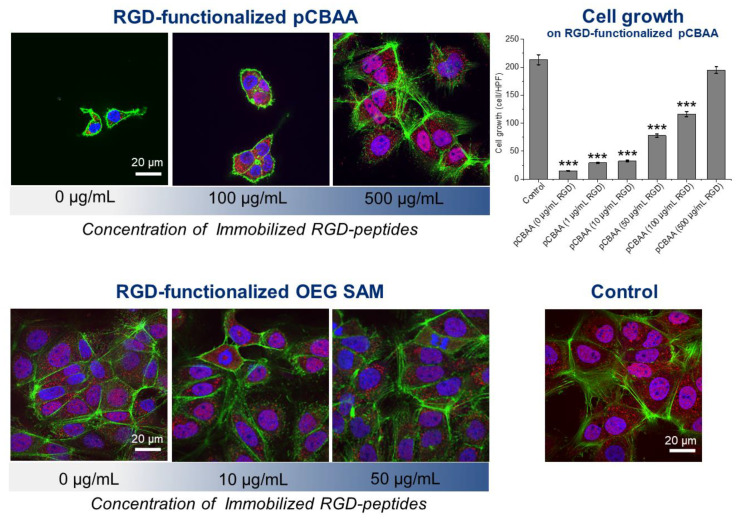
Growth of Huh7 cells on RGD-functionalized coatings. Examples of spinning disk confocal microscopy images of coatings functionalized by solutions of different concentrations of RGD peptides. Staining: Yes-associated protein (YAP) (red), F-actin (green), and nucleus (blue). Upper line: RGD-functionalized poly(carboxybetaine acrylamide) (pCBAA) functionalized using the method A-1 in Figure 1 (0, 100, and 500 µg/mL of the RGD peptides in the solution were used for functionalization) and plot of the dependency of the cell growth (number of cells per high-power field) on the RGD peptide concentrations. One-way analysis of variance with the Newman-Keuls test was performed, and data are expressed as means ± SEM, *** *p* < 0.001. Bottom line: RGD-functionalized OEG SAMs functionalized using the method A-2 in Figure 1 (0, 10, and 50 µg/mL of the RGD peptides in the solution were used for functionalization; no dependency was found) and the control (cells seeded onto standard 35-mm Petri dishes).

To assess the influence of the antifouling background on monitoring of the mechanotransduction, we used the prepared variable sets of RGD-functionalized (using method A and method B in Figure 1) pCB- and OEG SAM-based coatings for Huh7 cell cultivations for three days. Figure 2 shows a significant difference between these coatings. While the functionalized antifouling pCBAA brushes show a clear dependency of cell growth on the RGD peptide-immobilized level, there is no dependency found for the OEG SAM coatings (Figure 2, Figure S5, and Figure S6). Regarding the comparison of the superior antifouling properties of pCB-based brush coatings and rather poor resistance of OEG SAM coatings (given in Table 1 and Figure S3), these results suggest the importance of a proper antifouling background for the studies of cell signaling pathways. Clearly, the pCB-based background diminished most of the nonspecific interactions between the surface and cell cultivation medium or cells themselves, so the number of spots for cell adhesion may be easily controlled. 

**Figure 3:**
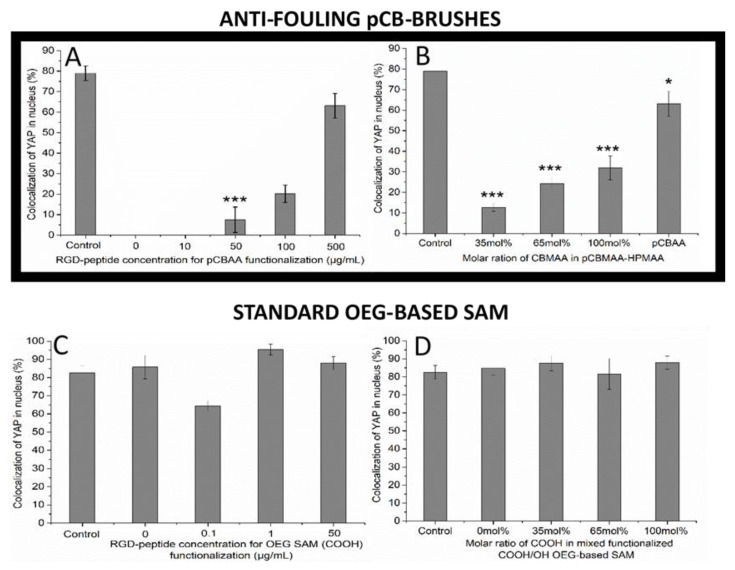
Colocalization of the YAP in the nucleus in cells growing on different RGD-functionalized surfaces. (**A**) Antifouling pCBAA coatings functionalized by solutions with different concentrations of RGD peptides (method A-1 in Figure 1). (**B**) Set of RGD-functionalized copolymer p(CBMAA-*ran*-HPMAA) antifouling coatings with increasing contents of functionalizable pCBMAA (method B-1 in Figure 1). (**C**) RGD-functionalized standard carboxy-ended OEG SAM (HS-(CH_2_)_11_-(EG)_6_-OCH_2_-COOH) (method A-2 in Figure 1). (**D**) RGD-functionalized mix of carboxy- and hydroxy-ended OEG SAM (HS-(CH_2_)_11_-(EG)_6_-OCH_2_-COOH and HS-(CH_2_)_11_-(EG)_4_-OH) (method B-2 in Figure 1). *** *p* < 0.001, * *p* < 0.05 mean significant differences with respect to control.

The adhesion number of cells around and the quality of the surface are key parameters directly influencing the mechanical stress-driven cell response. As a measurable parameter of such stress, the colocalization of YAP in the nucleus (“YAP colocalization” further) was determined for all sets of prepared functionalized coatings (Figure 3). The quantified YAP colocalization (shown in Figure 3C, D) shows no significant variation induced by different RGD-functionalized standard OEG SAM (functionalized using both method A-2 and A-3 according to Figure 1)-based coatings compared to the control surface. Apparently, such coatings are ineffective in the modulating of the YAP distribution, as they are ineffective in the modulation of cell growth and adhesion. Additionally, regarding such great insensitivity, it is not surprising that the functional groups (-COOH, -OH, and -RGD or a combination of them) show no effect on the cell stress response and YAP distribution.

On the other hand, the quantified YAP colocalization presented in Figure 3A shows a significant increase of the YAP level starting from 50 µg/mL of RGD peptide concentrations used to functionalize pCBAA brushes (method A-1 in Figure 1). The YAP colocalization reached almost the same level as on the control surface when the pCBAA brushes were functionalized with the concentration of 500 µg/mL of the RGD peptide. However, it is important to emphasize that there is a threshold of the amount of RGD peptide-functionalized spots on the surface, which is needed to observe the YAP colocalization changes (Figure 3A), despite the fact that the cell growth is tightly related to the RGD peptide concentration, even under such a threshold (Figure 2). Hypothetically, such observation may reveal the limiting “point of break” measure of mechanical stress given by the amount of accessible adhering spots and number of cells around at which cells start or stop the signaling pathway, changing the YAP distribution. 

Figure 3B shows YAP colocalization using the set of RGD-functionalized antifouling copolymer coatings (method B-1 in Figure 1). Obviously, the content of CBMAA in the copolymer brushes that correlates with RGD motif abundance modulates the YAP colocalization. However, even the RGD-functionalized homopolymer of 100% pCBMAA (functionalizable part of the copolymer brushes) did not reach the level of YAP colocalization induced by the control or the RGD-functionalized homopolymer of pCBAA (Figure 3B). In our previous study, the sensitivity of cell growth and spreading on the surface swelling characteristics was noticed, causing the higher growth and cytoskeleton distribution of cells growing on the nonfunctionalized pCBAA compared to pCBMAA [31]. Such a phenomenon can explain the different responses of the cells on functionalized pCBAA (method A-1 in Figure 1) and pCBMAA (method B-1 in Figure 1) and opens new possibilities in the design of coatings for the even finer modulation of cell growth and cell-signaling pathways.

## 4. Discussion

In the last decades, diverse polymer brushes with a variety of physicochemical properties and different abilities to control cell adhesion have been synthesized [4,38,39,40,41]. In the framework of these studies, we have prepared two different sets of RGD-functionalized antifouling coatings based on a zwitterionic pCBAA homopolymer or p(CBMAA-*ran*-HPMAA) copolymers with different contents of CBMAA. Our results demonstrate that, with both types of antifouling coatings, we are not only able to control the cell adhesion via specific integrin receptors but, also, to modulate the cell mechanotransduction signaling pathways. The results of the functionalized pCB-based polymer brushes were also compared with the corresponding RGD-functionalized standard OEG-based SAM coatings (carboxy-terminated or a mixture of carboxy- and hydroxy-terminated SAM). All OEG SAM coatings were ineffective in any modulation of cell adhesion, growth, or mechanotransduction.

The pCB-based polymer brushes exhibit superior antifouling and functionalization properties over numerous types of antifouling functionalizable coatings [34,42]. To achieve comparable RGD peptide immobilized levels on pCB coatings and OEG SAMs, the optimized functionalization procedures described in [42,43] were used and slightly modified to get sets of different surface concentrations of RGD peptides (Figure 1). In-line with previously reported studies [34,43,44], the antifouling character of the pCB brushes was not notably impaired, even after functionalization by RGD peptides. Even though OEG SAM coatings are considered as resistant to fouling from single-protein or diluted biological media, complex solutions challenge their resistance significantly [42,43]. Indeed, comparing foulings from undiluted blood plasma (Figure S3) or cell growth medium (Table 1) on functionalized coatings highlighted the extraordinary resistance of pCB brushes compared to standard OEG SAM coatings. Importantly, the comparison of the data from Figure 3 and Table 1 indicates that only small amounts of adhered materials nonspecifically adsorbed from the cell growth medium to the surface (~11.5 ng/cm^2^) may promote cell-surface interactions significantly. It is worth mentioning that interactions between the cells and OEG SAM substrate itself can contribute to the resulted inefficiency in any modulation of cell behavior. However, it is impossible to separate these two contributions in the used system.

A proper antifouling background appears to be a key parameter to ensure control over the abundance of spots of adhesion and enable the fine-tuning of the mechanically induced stress responses of cells. The control of the YAP distribution in the cell is a complex process influenced by many contradictory factors. Here, we report an increasing concentration of YAP in the nucleus with an increasing RGD motif surface coverage and increasing adhered cell number on functionalized antifouling surfaces. In [17,45], the authors reported decreasing YAP concentrations in the nucleus with an increasing cell population. On the other hand, according to the literature, more round geometry promotes the YAP localization in the cytoplasm. The more cells are spread, the more YAP is activated and localized in the nucleus [14,46]. The pCB-based antifouling coatings without any RGD moiety are not preferable surfaces for adhesion; cells tend to have rather round shapes compared to the standard controls [31,47]. The more spots of adhesion that are presented, the more cells are spreading on the surface, increasing the amount of YAP in the nucleus (Figure 2, Figure S5, and Figure 3). Obviously, in the case of homopolymer pCBAA coatings, there is no remarkable influence of other surface physicochemical properties, such as roughness, thickness, or hydration capabilities, as they do not change across the set of RGD-functionalized samples significantly. Clearly, in the case of pCBAA coatings with a limited number of spots of adhesion, the biggest contribution to YAP distribution-controlling factors have shape-driven factors. It is highly likely, that for even a higher number of adhered cells, the effect of a contact inhibition of proliferation [45,48] would be more significant, and we would observe the decreasing of YAP in the nucleus subsequently. 

In the case of copolymer brush coatings (method B-1 in Figure 1), one can consider even changes of the physicochemical properties of the surface, such as different hydrophilicity or the contents of zwitterionic moieties with changing ratios of the HPMAA content. Nevertheless, we observed the same dependency of YAP localization on the RGD peptide concentration (represented by a pCBMAA ratio in the copolymer), as on the pCBAA coating prepared by method A-1 in Figure 1.

It is worth mentioning that observed results should not be attributed to differences in surface stiffness. In our study, the polymer brushes having a wet thickness of ~80 nm on gold-coated glass substrates were used. It was reported previously that, for example, Petri dish substrates coated with polymers (e.g., PDMS) should be of about 40–200 μm thick to make a reliable change in the substrate stiffness for cell culturing [49,50,51].

YAP colocalization in the nucleus in cells grown on RGD-functionalized homopolymers pCBAA (method A-1 in Figure 1) and pCBMAA (method B-1 in Figure 1) differ remarkably, even though both coatings exhibit antifouling properties, even after RGD-peptide functionalization. In [31], we showed a higher tendency of F-actin cytoskeleton-spreading on pCBAA in comparison to pCBMAA. In the literature, the connection between F-actin polymerization and YAP nuclear concentration is noted—when F-actin polymerization happens, the nuclear level of YAP increases [45,52]. This is in accordance with our results shown in Figure 3B, and it underlines the influence of physicochemical properties of the surface composition on the cell behavior (even if it is nontoxic and antifouling). 

It is worth mentioning that we are aware of the limitations of this study that are related to a lack of quantification of RGD-peptide immobilization levels on pCB-based brushes. Using the available methods (for more details, see also the Supplementary Materials), a direct determination was found to be quite challenging. For example, while the level of immobilized RGD peptides on OEG SAM was quantified by the SPR method, it was not possible to determine the RGD-peptide surface concentration on the pCB-based coatings due to a very high pCB-based zwitterionic surface hydrophilicity [33,39,53] combined with a relatively small molecular weight of the RGD peptide (Figure S2 in the Supplementary Materials). Therefore, we were not able to directly compare the level of functionalization of pCBAA, pCBMAA-based coatings, and OEG-based coatings. However, in [42], we showed that the loading capacity of pCBMAA is quite comparable to pCBAA, so the comparable surface concentration of the RGD peptide can be expected on pCBMAA and pCBAA. Additionally, it should be noted that comparable protein-loading capacities for pCB-based brushes and OEG SAM were reported previously [43,54]. These findings indicate comparable saturation RGD peptide values for the respective functionalized coatings used in this study.

Zwitterionic materials and, namely, pCB-based materials represent emerging and very promising next-generation biomaterials for a wide range of biomedical applications [55]. Due to the excellent nonfouling properties and functionalization capabilities, such materials, in general, can be utilized to create various biomimetic systems, as well as multifunctional nanoparticles for multiple functional tissue scaffolds with cell-adhesive moieties and growth [56,57,58]. Therefore, the biomedical applications of zwitterionic functional materials continue to be a subject of extensive research and development.

## 5. Conclusions

Here, the RGD-functionalized pCB-based brushes were shown to be suitable and easily tunable platforms for mechanotransduction controlling and cell response manipulation. The results highlight the antifouling background as an essential element of cellular response studies. It was shown that zwitterionic-based antifouling brush architectures should be considered for the advanced design of next-generation surface platforms for the monitoring and quantification of cellular processes.

## Figures and Tables

**Table 1:** Fouling from the cell growth medium containing 10% fetal bovine serum on arginine, glycine, and aspartic acid (RGD)-functionalized coatings. The incubation time was 60 min; more experimental details are provided in the Supplementary Materials.

RGD Peptide Concentrations Used for Immobilization (µg/mL)	Fouling on pCBAA (ng/cm^2^)	RGD Peptide Concentrations Used for Immobilization (µg/mL)	Fouling on OEG-Based SAM (ng/cm^2^)
0	Undetectable ^1^	0	20.1
1	Undetectable ^1^	0.1	32.7
50	Undetectable ^1^	1	25.0
500	Undetectable ^1^	50	11.5

^1^ The value below the detection limit of the gold-coated glass substrates (SPR) (≈0.1 ng/cm^2^). pCBAA: poly(carboxybetaine acrylamide) and OEG SAM: oligo(ethylene glycol) self-assembled monolayer.

## Supplementary Materials

The following are available online at https://www.mdpi.com/2218-273X/10/8/1146/s1. Figure S1: Examples of OEG SAM RGD-functionalization (A) and CB-based polymer brush RGD-functionalization (B) measured by SPR. Figure S2: Dependence of the RGD-immobilized level (given by SPR) on the volume concentration of RGD in a functionalization solution (A,B) and on the carboxy group content in the coatings (B,D). (A) 100% OEG-based SAM HS-(CH2)11-(EG)6-OCH2-COOH, (B) polymer brush of 100% pCBAA, (C) mixed OEG SAM of HS-(CH2)11-(EG)6-OCH2-COOH and HS-(CH2)11-(EG)4-OH, and (D) random copolymer brush of p(CBMAA-ran-HPMAA). Figure S3: Fouling level from undiluted human blood plasma after 10 min of exposure on RGD-functionalized surfaces. Figure S4: Cell death assessment on pCB antifouling RGD-functionalized coatings. Figure S5: Representative pictures from the spinning disk confocal microscope used for the cell growth assessment and images of YAP distributions in cells growing on a CB-containing antifouling background decorated with different RGD peptide concentrations.
