# Supplementary material for: Functionalizable Antifouling Coatings as Tunable Platforms for the Stress-Driven Manipulation of Living Cell Machinery
Source: Biomolecules. 2020 Aug 5;10(8):1146. doi: 10.3390/biom10081146 (PMC7464033; doi:10.3390/biom10081146)
Supplement: Supplementary file 1 [file biomolecules-10-01146-s001.pdf]

Functionalizable Antifouling Coatings as Tunable Platforms for the Stress-Driven Manipulation of Living Cell Machinery

Ivana Víšová [1,†], Barbora Smolková [1,†], Mariia Uzhytchak [1,†], Markéta Vrabcová [1], Djamel Eddine Chafai [1], Milan Houska [1], Matěj Pastucha [2], Petr Skládal [2], Zdeněk Farka [2,*], Alexandr Dejneka [1] and Hana Vaisocherová-Lísalová [1,*]

[1] Institute of Physics CAS, Na Slovance 1999/2, 182 21, Prague, Czech Republic; visova@fzu.cz (I.V.); smolkova@fzu.cz (B.S.); uzhytchak@fzu.cz (M.U.); vrabcova@fzu.cz (M.V.); chafai@fzu.cz (D.E.C.); houska@fzu.cz (M.H.); dejneka@fzu.cz (A.D.)

[2] Department of Biochemistry, Faculty of Science, Masaryk University, Kamenice 5, 625 00, Brno, Czech Republic; mpastucha@gmail.com (M.P.); skladal@chemi.muni.cz (P.S.)

* Correspondence: farka@mail.muni.cz (Z.F.); lisalova@fzu.cz (H.V.-L.); Tel.: +420549497674 (Z.F.); +420266052993 (H.V.-L.)

† These authors contributed equally to this work.

SPR characterization of functionalization and antifouling properties:

Functionalization of OEG-based AT SAM coatings: Chips were rinsed with ethanol, ultrapure water, and mounted to the SPR sensor. In the sensor, coatings were washed by ultrapure water for 10 min and subsequently activated with a solution of 0.025 M NHS + 0.125 M EDC for 10 min. Afterwards, coatings were shortly washed with 10 mM sodium acetate buffer (pH 5.0) (SA10) and the solution of RGD-peptide in SA10 was injected (Figure S1). For coatings of pure HS-$(CH_2)_{11}$-$(EG)_6$-OCH_2-COOH, immobilization solutions of different concentrations of RGD-peptide were used (0.1, 1, 50, 100 μg/mL). For immobilization of RGD-peptide on mixed coatings with different ratios of HS-$(CH_2)_{11}$-$(EG)_6$-OCH_2-COOH : HS-$(CH_2)_{11}$-$(EG)_4$-OH, a concentration of 50 μg/mL was applied. After 12 min of immobilization, sodium acetate buffer was injected to form a baseline. The level of immobilized peptide is set as the difference between sodium acetate buffer baseline before and after the immobilization step (Figure S1 A).

Functionalization of CB-based coatings: Chips were rinsed with ultrapure water and mounted to the SPR sensor. In the sensor, the coatings were washed by ultrapure water for 10 min and subsequently activated with a solution of 0.1 M NHS + 0.5 M EDC for 25 min. Afterwards, coatings were shortly washed with ultrapure water, and the solution of RGD-peptide in 10 mM borate buffer (pH 8) was injected (Figure S1) For pCBAA and pCBMAA coatings, immobilization solutions of different concentrations of RGD-peptide were used (1, 10, 100, 500 μg/mL). For immobilization of RGD-peptide on copolymers p(CBMAA-*ran*-HPMAA) concentration of 850 μg/mL was applied. After 20 min of immobilization, ultrapure water was injected to form a baseline. The level of immobilized peptide is set as a difference between the ultrapure water baseline before and after the immobilization step (Figure S1 B).

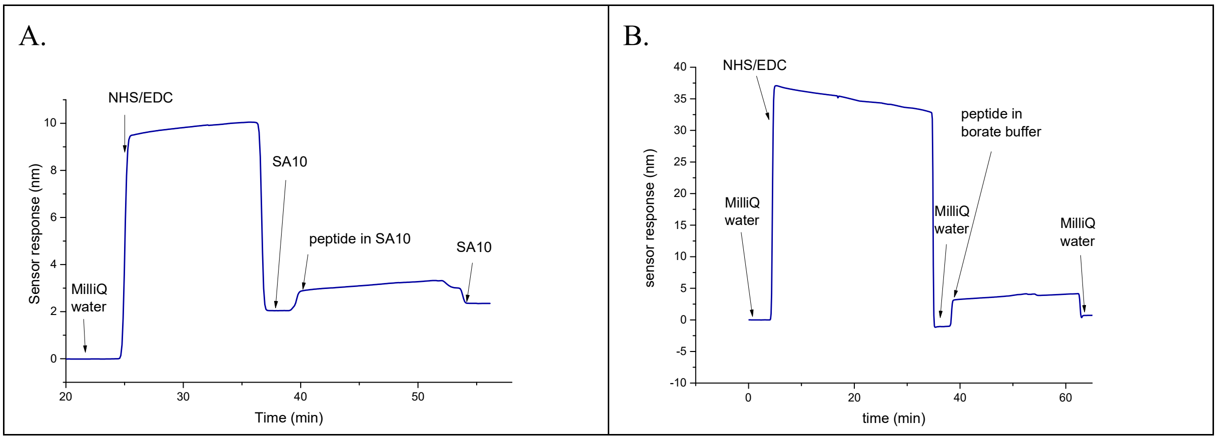

Figure S1: Examples of OEG SAM RGD-functionalization (A.) and CB-based polymer brush RGD-functionalization (B.) measured by SPR

The coatings were characterized by SPR to confirm the dependence of surface coverage on the volume concentration of RGD in the functionalization solution. Figures S2 A and C show the expected dependence for OEG-based SAM coatings. Unfortunately, due to the high degree of swelling of the polymer brush coatings [9], the principle of SPR method, and the small weight of peptide, there is no clear dependency found on the polymer brush coatings (Figure S2 B and D). Due to the nature of the system (high hydrophilicity of pCB brushes, small weight of peptide, the complexity of pCB brush structures and amino-coupling chemistry used for RGD-peptide immobilization) the quantification was not also possible based on data obtained from spectroscopic ellipsometry, infrared reflection spectroscopy or ninhydrin assay.

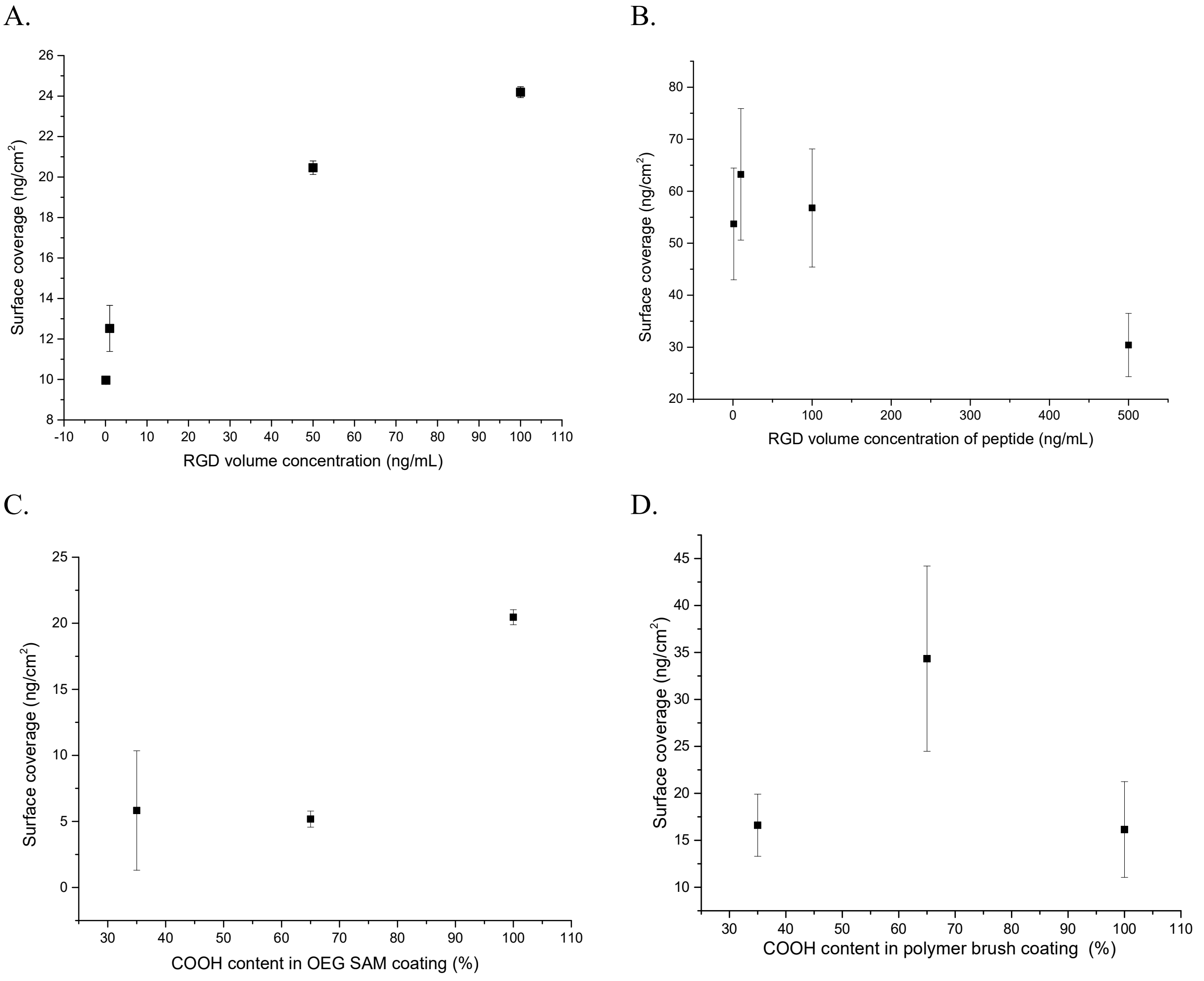

Figure S2: Dependence of RGD-immobilized level (given by SPR) on volume concentration of RGD in functionalization solution (A, B) and on carboxy-group content in coatings (B, D). A: 100% OEG based SAM HS-$(CH_2)_{11}$-$(EG)_6$-OCH_2-COOH, B: polymer brush of 100% pCBAA, C: mixed OEG SAM of HS-$(CH_2)_{11}$-$(EG)_6$-OCH_2-COOH and HS-$(CH_2)_{11}$-$(EG)_4$-OH, D: random copolymer brush of p(CBMAA-ran-HPMAA).

Fouling characterization: Functionalized chips prepared according to the procedures described above were incubated in PBS for 10 min. Afterwards, undiluted human blood plasma or growth medium (EMEM medium (ATCC) supplemented with 10% fetal bovine serum) were injected for 10 min or 60 min, respectively. Subsequently, the chips were washed by PBS for 10 min, followed by higher ionic strength PBS-NaCl for 5 min, completed by relaxation in PBS for another 10 min. Fouling level was assessed as the difference between

PBS baseline before plasma injection and after 10 min of PBS after higher ionic strength PBS washing step. The results are shown in Table 1.

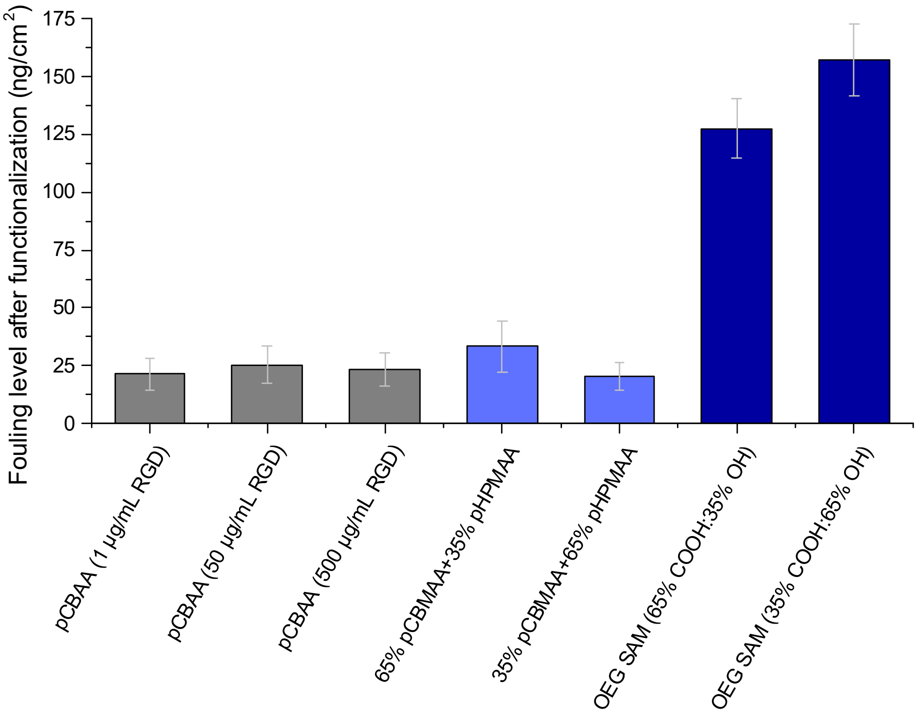

Figure S3: Fouling level from undiluted human blood plasma after 10 min of exposure on RGD-functionalized surfaces.

Cell death assessment: Quantitative analysis of the viability of Huh7 cells on different pCB ultra-low fouling functionalized coatings after 72 h of cultivation. Cells were labeled with propidium iodide (PI in red) and Hoechst (blue) was used for nuclear staining. PI-positive cells were considered to be dead cells. Labeled cells were imaged using spinning disk confocal microscopy (Spin SR, Olympus). ImageJ software (NIH) was used for image processing and quantification. One-way ANOVA with Newman–Keuls multiple comparison test was used. Data are expressed as means ± SEM (n = 3), ***P< 0.001. As a ”positive control”, cells were incubated with 20% ethanol. “Control” cells were seeded onto standard 35-mm Petri dishes.

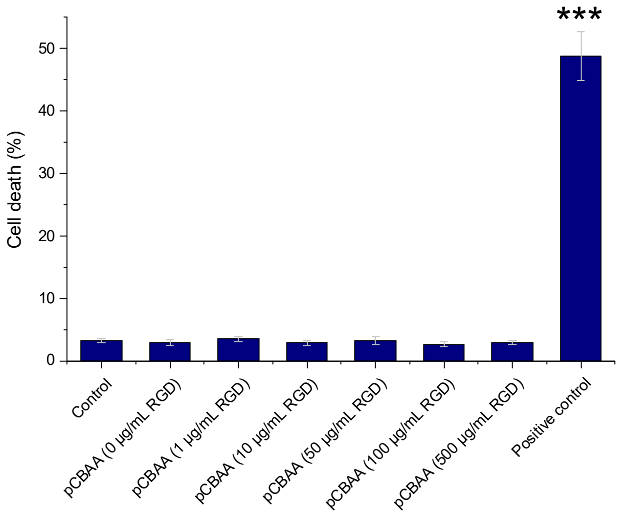

Figure S4: Cell death assessment on pCB antifouling RGD-functionalized coatings.

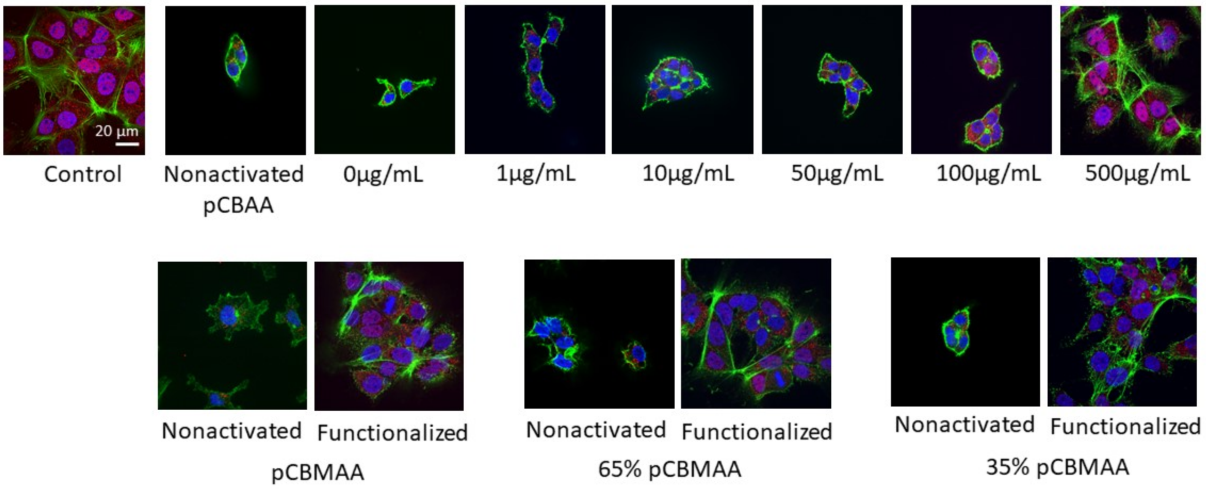

Figure S5: Representative pictures from spinning disk confocal microscope used for cell growth assessment and images of YAP distribution in cells growing on CB-containing antifouling background decorated with different RGD-peptide concentrations. Cells were stained with the YAP antibody in red, F-actin in green, and Hoechst (blue) was used for nuclear staining. Upper line: Examples of cells growing on the control surface and surfaces prepared by the method described in Section 3.1, Figure A1. Concentrations of RGD-peptide in solutions used for immobilization after surface activation are marked under each image. Bottom line: Examples of cells growing on the surfaces prepared by the method described in Section 3.1, Figure B1. As „nonactivated", bare NHS/EDC non-activated surfaces without immobilized peptide are marked. „Functionalized" surfaces were activated and the solution of 850 µg/mL of RGD-peptide was used for the immobilization.

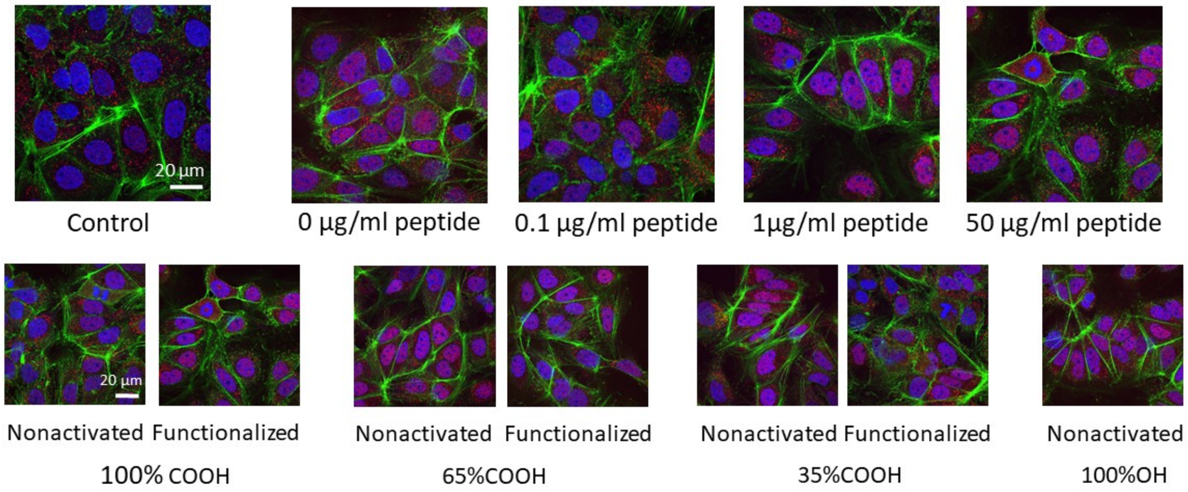

Figure S6: Representative pictures from spinning disk confocal microscope used for cell growth assessment and images of YAP distribution in cells growing on OEG SAM background decorated with different RGD-peptide concentrations. Cells were stained with the YAP antibody in red, F-actin in green and Hoechst (blue) was used for nuclear staining. Upper line: Examples of cells growing on the control surface and surfaces prepared by the method described in Section 3.1, Figure A2. Concentrations of RGD-peptide in solutions used for immobilization after surface activation are marked under each image. Bottom line: Examples of cells growing on surfaces prepared by the method described in Section 3.1, Figure B2. As „nonactivated", bare NHS/EDC non-activated surfaces without immobilized peptide are marked. „Functionalized" surfaces were activated and the solution of 50 µg/mL of RGD-peptide was used for the immobilization.
